# Machine learning-based ozone and PM2.5 forecasting: Application to multiple AQS sites in the Pacific Northwest

**DOI:** 10.3389/fdata.2023.1124148

**Published:** 2023-02-24

**Authors:** Kai Fan, Ranil Dhammapala, Kyle Harrington, Brian Lamb, Yunha Lee

**Affiliations:** ^1^Center for Advanced Systems Understanding, Görlitz, Germany; ^2^Helmholtz-Zentrum Dresden Rossendorf, Dresden, Germany; ^3^Laboratory for Atmospheric Research, Department of Civil and Environmental Engineering, Washington State University, Pullman, WA, United States; ^4^South Coast Air Quality Management District, Diamond Bar, CA, United States; ^5^Max Delbrück Center for Molecular Medicine, Berlin, Germany

**Keywords:** machine learning, air quality forecasts, ozone, PM2.5, random forest, multiple linear regression

## Abstract

Air quality in the Pacific Northwest (PNW) of the U.S has generally been good in recent years, but unhealthy events were observed due to wildfires in summer or wood burning in winter. The current air quality forecasting system, which uses chemical transport models (CTMs), has had difficulty forecasting these unhealthy air quality events in the PNW. We developed a machine learning (ML) based forecasting system, which consists of two components, ML1 (random forecast classifiers and multiple linear regression models) and ML2 (two-phase random forest regression model). Our previous study showed that the ML system provides reliable forecasts of O_3_ at a single monitoring site in Kennewick, WA. In this paper, we expand the ML forecasting system to predict both O_3_ in the wildfire season and PM2.5 in wildfire and cold seasons at all available monitoring sites in the PNW during 2017–2020, and evaluate our ML forecasts against the existing operational CTM-based forecasts. For O_3_, both ML1 and ML2 are used to achieve the best forecasts, which was the case in our previous study: ML2 performs better overall (R^2^ = 0.79), especially for low-O_3_ events, while ML1 correctly captures more high-O_3_ events. Compared to the CTM-based forecast, our O_3_ ML forecasts reduce the normalized mean bias (NMB) from 7.6 to 2.6% and normalized mean error (NME) from 18 to 12% when evaluating against the observation. For PM2.5, ML2 performs the best and thus is used for the final forecasts. Compared to the CTM-based PM2.5, ML2 clearly improves PM2.5 forecasts for both wildfire season (May to September) and cold season (November to February): ML2 reduces NMB (−27 to 7.9% for wildfire season; 3.4 to 2.2% for cold season) and NME (59 to 41% for wildfires season; 67 to 28% for cold season) significantly and captures more high-PM2.5 events correctly. Our ML air quality forecast system requires fewer computing resources and fewer input datasets, yet it provides more reliable forecasts than (if not, comparable to) the CTM-based forecast. It demonstrates that our ML system is a low-cost, reliable air quality forecasting system that can support regional/local air quality management.

## 1. Introduction

The AIRPACT air quality forecast system for the Pacific Northwest has been used for air quality forecasts in the Pacific Northwest (PNW) since May 2001 (Chen et al., 2008). AIRPACT uses Weather Research and Forecasting (WRF) meteorological model forecasts produced daily by the University of Washington as input to the Community Multiscale Air Quality (CMAQ) to simulate the air quality over the PNW. It provides detailed air quality forecasts, but requires considerable computational power, and the forecast accuracy is not satisfactory for poor air quality events. Our study on the decadal evaluation of AIRPACT forecast reveals that major updates made to the AIRPACT system during the past decade did not improve the forecast capability significantly (Munson et al., 2021). For instance, AIRPACT's skill has improved slightly over time for ozone (O_3_) but not for fine particulate matter (PM2.5). The PM2.5 predictions were largely under-predicted during the wildfire season in the years 2015 and 2018.

Machine learning models have been employed successfully to predict the air quality across regions in other studies (e.g., Yu et al., 2016; Kang et al., 2018; Rybarczyk and Zalakeviciute, 2018; Zhan et al., 2018; Pernak et al., 2019; Li et al., 2022). For example, Yuchi et al. (2019) utilized random forest regression and multiple linear regression models for indoor PM2.5 predictions. Zamani Joharestani et al. (2019) applied three models (i.e., random forest, extreme gradient boosting, and deep learning) to predict the PM2.5 concentrations in Tehran's urban area. Eslami et al. (2020) used a deep convolutional neural network to predict the hourly O_3_ across 25 observation stations over Seoul, South Korea. Xiao et al. (2020) and Liu and Li (2022) proposed two deep learning methods based on the Long-Short Term Memory (LSTM) neural network to predict the PM2.5 concentrations in the Beijing–Tianjin–Hebei region of China. Yang et al. (2021) explored the traffic impacts on air quality by a random forest model under the pandemic scenario in Los Angeles. Chau et al. (2022) applied deep learning methods, LSTM and Bidirectional Recurrent Neural Network, to study the effects of COVID-19 lockdown on the air quality change.

We developed an O_3_ forecasting system based on machine learning (ML) models to improve O_3_ predictions in Kennewick, WA during wildfire seasons in the PNW region (Fan et al., 2022). In Fan et al. (2022), we used a single monitoring site in Kennewick, WA during the wildfire seasons in 2017 to 2020. This ML system consists of two components, ML1 (random forecast classifiers and multiple linear regression models) and ML2 (two-phase random forest regression model); see Supplementary Figure 1 for the details of ML1 and ML2 components. In Fan et al. (2022), we found that our ML forecasts captured 50% of unhealthy O_3_ events in Kennewick, WA, which was a big improvement given that AIRPACT missed all of them.

In this paper, we expand the application of our ML modeling framework to all O_3_ and PM2.5 monitoring sites available in US EPA's Air Quality System (AQS) database throughout the PNW from 2017 to 2020. This study applies the ML system to predict O_3_ as well as PM2.5 forecasts, compared to only O_3_ in Fan et al. (2022). The goal of our study is to test our ML-based air quality forecast framework more rigorously by increasing spatiotemporal coverages of observations and to compare our ML-based forecasts to the CTM-based AIRPACT system.

The paper is organized as follows: Section 2 presents the input data, technical details of the ML forecast framework, and model validation methods. The subsequent result section (i.e., O_3_ in 2017 to 2020 Wildfire Seasons and PM2.5 in 2017 to 2020 Wildfire and Cold Seasons) present the evaluation of the ML model performance on O_3_ and PM2.5 predictions in the PNW using 10-time, 10-fold cross-validation. The last section provides a summary and conclusions.

## 2. Data and methods

### 2.1. ML predictions of O_3_ and PM2.5 using observation datasets

In the PNW, currently there are 47 AQS sites with O_3_ observations, 138 sites with PM2.5 observations. Similar to the ML modeling framework for Kennewick, the training dataset for this multi-site ML models included the previous day's observed O_3_ or PM2.5 concentrations, time information (hour, weekday, month represented as factors), and hourly meteorological forecast data from twice-daily ensemble WRF forecasts extracted at each AQS site. The WRF meteorology data was provided by the twice-daily ensemble forecasts with 4 km horizontal resolution, produced by the University of Washington (UW, https://a.atmos.washington.edu/mm5rt/ensembles/).

The UW ensemble system applies multiple physical parameterizations and surface properties to the WRF model simulations, and the ensemble forecasts could improve the forecast skill for some cases (Grimit and Mass, 2002; Mass et al., 2003; Eckel and Mass, 2005). To utilize the varying settings for the meteorology simulations, we input the multi-member WRF ensemble forecasts for the air quality forecasts in the PNW.

The evaluation of O_3_ predictions in this paper covers May to September from 2017 to 2020 and PM2.5 predictions cover two seasons, wildfire season (May to September) and cold season (November to February) from 2017 to 2020. While wildfires can affect both O_3_ and PM2.5 concentrations significantly, wood burning from stoves during cold season is a significant source of PM2.5 in populated areas, so we look at only PM2.5 for cold season. To identify the characteristics of each individual site, the models are trained for each monitoring site with archived 4 km WRF forecasts and observations. For the model evaluation, we used the archived operational WRF data, which is a single ensemble WRF member from UW forecasts. The observations and archived WRF data are available at 30 sites for O_3_ and more than 100 sites for PM2.5, and there are 12 sites where both O_3_ and PM2.5 are measured.

### 2.2. Machine learning modeling framework

We developed an ML-based air quality forecast modeling framework that consists of two independent ML models, in order to predict O_3_ at Kennewick, WA (Fan et al., 2022). The first ML model (ML1; Supplementary Figure 1a) consists of a random forest classifier and a multiple linear regression model: the *RandomForestClassifier* and *RFE* functions in the Python library *scikit-learn* are used (Pedregosa et al., 2011). The second ML model (ML2; Supplementary Figure 1b) is based on a two-phase random forest regression model: the *RandomForestRegressor* function in the Python library *scikit-learn* is used (Pedregosa et al., 2011). More details of our ML modeling framework are available in Dataset and Modeling Framework section in Fan et al. (2022).

In this study, we use the same ML models to predict the O_3_ and PM2.5 at various AQS sites in the PNW. To better fit the local conditions, the model is trained at each individual site. Hourly O_3_ and PM2.5 predictions are used to compute maximum daily 8-h running average (MDA8) O_3_ mixing ratio and 24-h averaged PM2.5 concentrations, as these are the requirements of the National Ambient Air Quality Standards (NAAQS). Due to the different sources of PM2.5 during wildfire and cold seasons in the PNW, the model is trained separately for two seasons at each site. The feature-selection module from the functions listed above are used to select the features at each site to train the models. For ML2, the weighting factors vary at each site, which are computed based on the local input data.

Given ML models can be subject to overfitting and can be sensitive to issues in the training dataset, we account for these issues in our modeling setup. To avoid overfitting, we limit five features in the model training, and use 10-time 10-fold cross-validation to evaluate our model. Our training datasets are air quality observation, which are generally imbalanced: a highly polluted event or an extremely clean event is a rare event. Haixiang et al. (2017) shows that imbalanced training data may lead a bias toward commonly observed events. To alleviate the imbalance problem, we apply several methods such as turning on the *balanced_subsample* option in the function of the random forest model and using multiple linear regression and second phase random forest regression in the modeling system.

### 2.3. Computational requirements

Our ML modeling framework requires much less computational power than the AIRPACT CMAQ system. Whereas AIRPACT requires approx. 360 h of CPU time (120 processors for ~3 h) for a single daily forecast, it takes 1–2 h of CPU time to run the ML model for the 25–30 member WRF ensemble O_3_ predictions at 47 AQS sites and PM2.5 at 138 AQS sites throughout the PNW using the same CPU resources (Intel Xeon E5-2620 v4). The exact number of WRF members may vary. The ML model is re-trained monthly using the averaged WRF ensemble forecasts at these sites and requires about 40 min of CPU time.

### 2.4. Validation method and evaluation metrics

We use three forecast verification metrics. Heidke Skill Score (HSS), a commonly used forecast verification metric, is used to evaluate the model predictability on AQI categories. Note that HSS represents the accuracy of the model prediction compared with a “random guess”-based forecast that is statistically independent of the observations, and the value less than 0 means no skill and the value close to 1 means a skillful model (Wilks, 2011; Jolliffe and Stephenson, 2012). Another forecast verification metric, Hanssen-Kuiper Skill Score (KSS), measures the ability to separate different categories: the value less than 0 means no skill and the value close to 1 means a skillful model (Wilks, 2011; Jolliffe and Stephenson, 2012). The Critical Success Index (CSI) score is the ratio of correct predictions to the total number of observed or forecast events at each category, whose range is from 0 to 1, and the closer to 1, the more skillful the model is at this category (Wilks, 2011; Jolliffe and Stephenson, 2012).

We also use a Taylor diagram to compare the model performance throughout the sites in the PNW (Taylor, 2001; Lemon, 2006). Three statistical variables, namely the standard deviation (SD), correlation coefficient (R), centered root-mean-square (RMS) difference, are shown in a Taylor diagram. They are computed based on Equations (1)–(4), where m and o refer to the model predictions and observations.


(1) $$\begin{array}{l} \sigma_{M}=\sqrt{\frac{1}{n}\overset{n}{\underset{i=1}{\sum }}{\left(m_{i}-\bar{m}\right)}^{2}} \end{array}$$

(2) $$\begin{array}{l} \sigma_{O}=\sqrt{\frac{1}{n}\overset{n}{\underset{i=1}{\sum }}{\left(o_{i}-ō\right)}^{2}} \end{array}$$

(3) $$\begin{array}{l} R=\frac{1}{\sigma_{O}\sigma_{M}}\frac{1}{n}\overset{n}{\underset{i=1}{\sum }}\left(m_{i}-\bar{m}\right)\left(o_{i}-ō\right) \end{array}$$

(4) $$\begin{array}{l} Centered\;RMS\;difference=\;\sqrt{\frac{1}{n}\overset{n}{\underset{i=1}{\sum }}{\left(\left(m_{i}-\bar{m}\right)-\;\left(o_{i}-ō\right)\right)}^{2}} \end{array}$$

The refined index of agreement (IOA) is used to compare the model accuracy, and its range is from −1 to 1 (Willmott et al., 2012). The IOA of a good model is close to 1. An R function *dr()* from the package *ie2misc* using Equations (5) and (6) is used to compute the IOA (Embry et al., 2022).


(5) $$\begin{array}{l} d_{r}=1-\frac{\sum_{i=1}^{n}\left|m_{i}-o_{i}\right|}{2\sum_{i=1}^{n}\left|o_{i}-\bar{o}\right|}\text{ when }\sum_{i=1}^{n}\left|m_{i}-o_{i}\right|\;\leq 2\sum_{i=1}^{n}\left|o_{i}-\bar{o}\right| \end{array}$$

(6) $$\begin{array}{l} d_{r}=\frac{\sum_{i=1}^{n}\left|m_{i}-o_{i}\right|}{2\sum_{i=1}^{n}\left|o_{i}-{\bar{o}}_{i}\right|}-1\text{ when }\sum_{i=1}^{n}\left|m_{i}-o_{i}\right|\;>2\sum_{i=1}^{n}\left|o_{i}-\bar{o}\right| \end{array}$$

## 3. O_3_ in 2017 to 2020 wildfire seasons

This study covers a typical wildfire season in the PNW region, from May to September, in 2017 to 2020 and uses O_3_ observations at 30 AQS sites in this region. Table 1 summarizes the observed O_3_ average values and the number of monitor-days in each year with each AQI category that is computed only with the maximum daily 8-h running average (MDA8) O_3_ mixing ratio. The number of monitor-days in each year is presented in the parenthesis. The MDA8 O_3_ observations in this region are mostly within lower levels: “Good” air with an AQI category of 1 (76.6 to 90.8% of total days used in this study) and “Moderate” air with an AQI category of 2 (8.8 to 20.1% of total days used in this study). There is an annual variability in O_3_ during this period. For example, the O_3_ means are higher in 2017 and 2018 (44 and 43 ppb) than in 2019 and 2020 (39 and 40 ppb). Also, the number of monitor-days where air quality was “Unhealthy for Sensitive Groups” (AQI_3_) and “Unhealthy” (AQI_4_) are noticeably more frequent in 2017 and 2018, which could be attributed to more wildfires during these years. It is very important to predict these unhealthy events reliably as an air quality forecasting system, but AIRPACT operational air quality forecasting system failed to predict all 14 unhealthy O_3_ events (AQI_4_) during the wildfire seasons of 2017 to 2020.

**Table 1:** Summary of the O_3_ observations from May to September in 2017 to 2020 at 30 AQS sites in the PNW region. Note that daily AQI is computed using MDA8 O_3_ only.

**Year**	**Mean (ppb)**	**Percentage and # of monitor-days for each AQI category**				
		**1**	**2**	**3**	**4**	**Total**
2017	44	76.6% (2,971)	20.1% (778)	3.1% (120)	0.2% (9)	3,878
2018	43	78.3% (3,195)	19.8% (808)	1.8% (75)	0.1% (4)	4,082
2019	39	90.8% (3,728)	8.8% (361)	0.4% (15)	0 (0)	4,104
2020	40	88.8% (3,361)	10.3% (390)	0.9% (35)	0 (1)	3,787

### 3.1. Evaluating O_3_ predictions of AIRPACT and ML models

The 10-time, 10-fold cross-validation is used to evaluate the model performance throughout the AQS sites over the PNW region. Our forecast values are initially hourly but compiled into MDA8 O_3_, and then we compare our ML performance against the CTM-based air quality forecasting system, AIRPACT.

**Figure 1:**
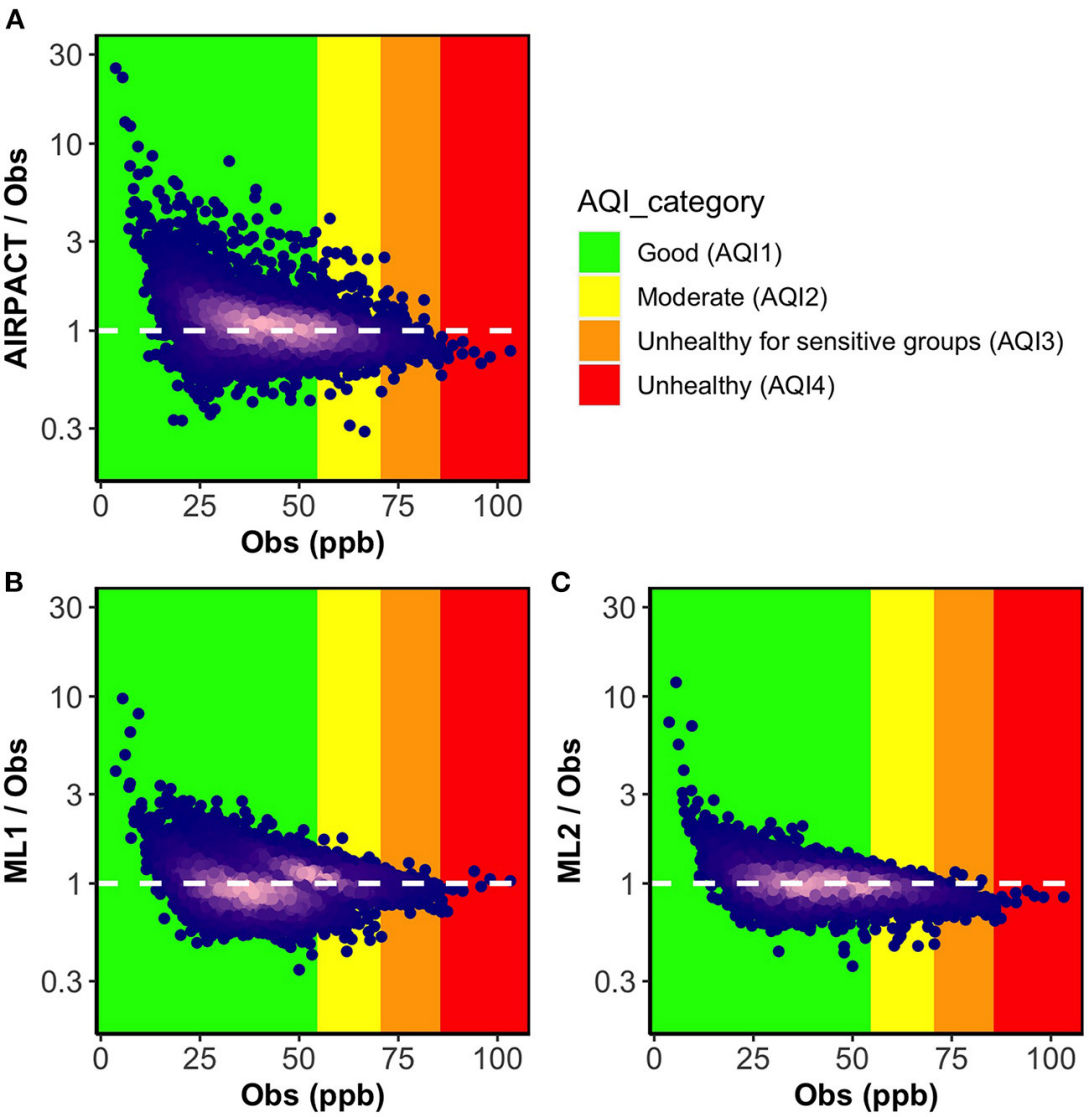
Ratio plots of model predicted MDA8 O_3_ to observations vs. observations for three models **(A)** AIRPACT, **(B)** ML1, and **(C)** ML2. The point color of dark blue to bright pink indicates density of the data increasing. The white dashed lines mark the ideal condition (the ratio between model predictions and observations is 1). The ratio below 1 represents the model under-prediction and the ratio above 1 represents the over-prediction.

To examine how the model performance varies by O_3_ levels, we present the ratio plots of simulated to measured MDA8 O_3_ against the measured MDA8 O_3_ levels from the 30 AQS sites in Figure 1. The densest parts of the data in bright pink are near the ratio of 1, which indicates most of the predictions are close to the observations. All models have a similar issue that over-predictions seem to be worse at lower O_3_ levels. For AIRPACT, the model-to-observation agreement is noticeably more scattered across the O_3_ levels than the ML models, which leads to extremely under-predicted or over-predicted MDA8 O_3_ forecasts that result in more misses or false alarms during the operational forecasting. For instance, AIRPACT predicts 1% of good air quality events as unhealthy for sensitive groups (i.e., false alarms) and 7% of unhealthy air quality events as good (i.e., misses; see Supplementary Figure 2). For the ML models, extremely incorrect predictions are fewer than AIRPACT. Compared to ML1, ML2 agrees better with observation as it shows the least scattered MDA8 O_3_ distribution along with the O_3_ levels. We can also see that the densest part of the data is over the AQI_1_ (green) and AQI_2_ (yellow) categories in Figure 1C, where more than 95% of the O_3_ observations used in this study fall into, is very close to the ratio of 1. For the higher O_3_ events (i.e., AQI_3_ and AQI_4_), ML2 under-predicts most of these events, which is concerning as correctly forecasting a high-O_3_ events is crucial to support air quality-related public health.

The model evaluation statistics of MDA8 O_3_ at 30 AQS sites over the PNW region during 2017 to 2020 are summarized in Table 2. All ML models outperform AIRPACT, and ML2 is the best among the ML models: ML2 has R^2^ of 0.79, NMB of −0.68%, NME of 11%, and IOA of 0.79, while AIRPACT has R^2^ of 0.42, NMB of 7.6%, NME of 18%, and IOA of 0.64.

**Table 2:** Statistics of the 10-time, 10-fold cross-validation of the MDA8 O_3_ predictions from AIRPACT and our ML models.

	**AIRPACT**	**ML1**	**ML2**	**ML_opr_O_3_**
R^2^	0.42	0.67	0.79	0.76
NMB (%)	7.6	2.2	−0.68	2.6
NME (%)	18	16	11	12
IOA	0.64	0.69	0.79	0.76

The model evaluations using HSS and KSS forecast verification metrics are based on the AQI computed with only O_3_ from each model and are presented in Table 3. Similar to the statistics in Table 2, all ML models show higher HSS and KSS scores than AIRPACT. For HSS, ML2 has a higher score (0.59) than ML1 (0.47). For KSS, ML1 has a higher score (0.61) than ML2 (0.55), because ML1 distinguishes the AQI categories better by predicting more days with AQI_3_ and AQI_4_ categories than ML2.

**Table 3:** Forecast verifications of the 10-time, 10-fold cross-validations using AQI computed with only O_3_ from AIRPACT and our ML models.

		**AIRPACT**	**ML1**	**ML2**	**ML_opr_O_3_**
HSS		0.46	0.47	**0.59**	0.54
KSS		0.49	0.61	0.55	**0.63**
CSI	1	0.83	0.80	**0.89**	0.86
	2	0.36	0.36	**0.46**	0.41
	3	0.16	**0.21**	0.038	0.21
	4	0	0.062	**0.12**	0.062

The best statistical values are marked with bold fonts.

The CSI in Table 3 measures the model's AQI categorical forecast. ML2 has the highest CSI_1_ (0.89) and CSI_2_ (0.46) score, and ML1 has the highest CSI_3_ score (0.21), which is consistent with what we see in Figure 1. However, the CSI_4_ score of ML1 (0.062) is lower than ML2 (0.12), despite the number of AQI_4_ events captured by ML1 and ML2 are same (see Supplementary Figure 2). This is because ML1 tends to predict higher O_3_ levels than ML2 (see Figures 1B, C), which leads to more “false” AQI_3_ and AQI_4_ predictions. For a very rare event such as AQI_4_, the CSI score is significantly influenced by having a few more false alarms.

**Figure 2:**
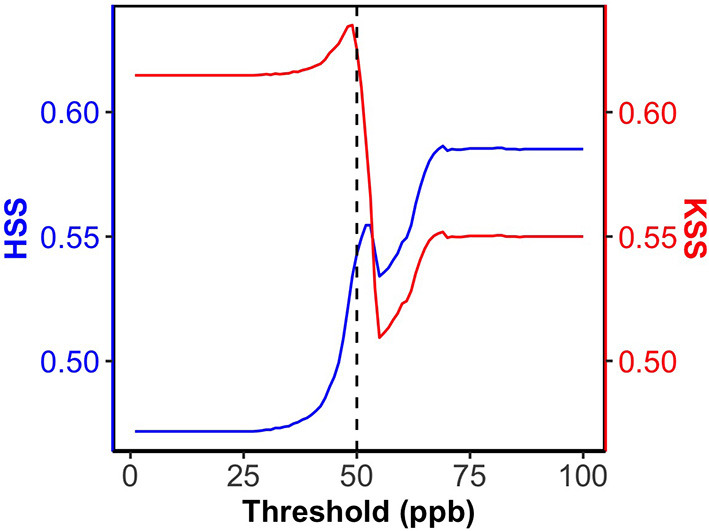
The evaluations of HSS and KSS with increasing the threshold.

In order to produce the most reliable O_3_ predictions with our ML models, we build an operational ML modeling framework for O_3_ to use ML2 for low O_3_ levels and ML1 for high O_3_ levels (“ML_opr_O_3_” in Tables 2, 3). The ML_opr_O_3_ model requires a threshold O_3_ level that determines which ML prediction (ML1 or ML2) to be as a final forecast product. If the ML2 prediction is lower than the threshold, then the ML2 prediction is selected; if not, the ML1 prediction is selected. To find an optimal threshold O_3_ level that enables either ML1 or ML2, we tested the threshold value from 1 to 100 ppb and computed the evolutions of HSS and KSS (see Figure 2). A low threshold means more ML1 predictions are used. With increasing the threshold value, more ML2 predictions are used and the HSS value is increased. This is consistent with the high HSS value from ML2 in Table 2. When the threshold value is above 50 ppb, the increasing trend of HSS stops and the KSS value dramatically decreases. Thus, ML_opr_O_3_ uses ML2 when MDA8 O_3_ predictions by ML2 are less than 50 ppb and, otherwise, uses ML1. ML_opr_O_3_ performance is mostly in between ML1 and ML2 in Tables 2, 3. The statistics (R^2^, NME, IOA, HSS, CSI_1_, and CSI_2_) between ML2 and ML_opr_O_3_ is close as our O_3_ observation data is mostly for lower O_3_ levels where ML_opr_O_3_ relies on ML2. Using ML1 predictions improves the model performance (KSS and CSI_3_) for high O_3_ events, although some over-predictions lead to a higher NMB value than ML2.

**Figure 3:**
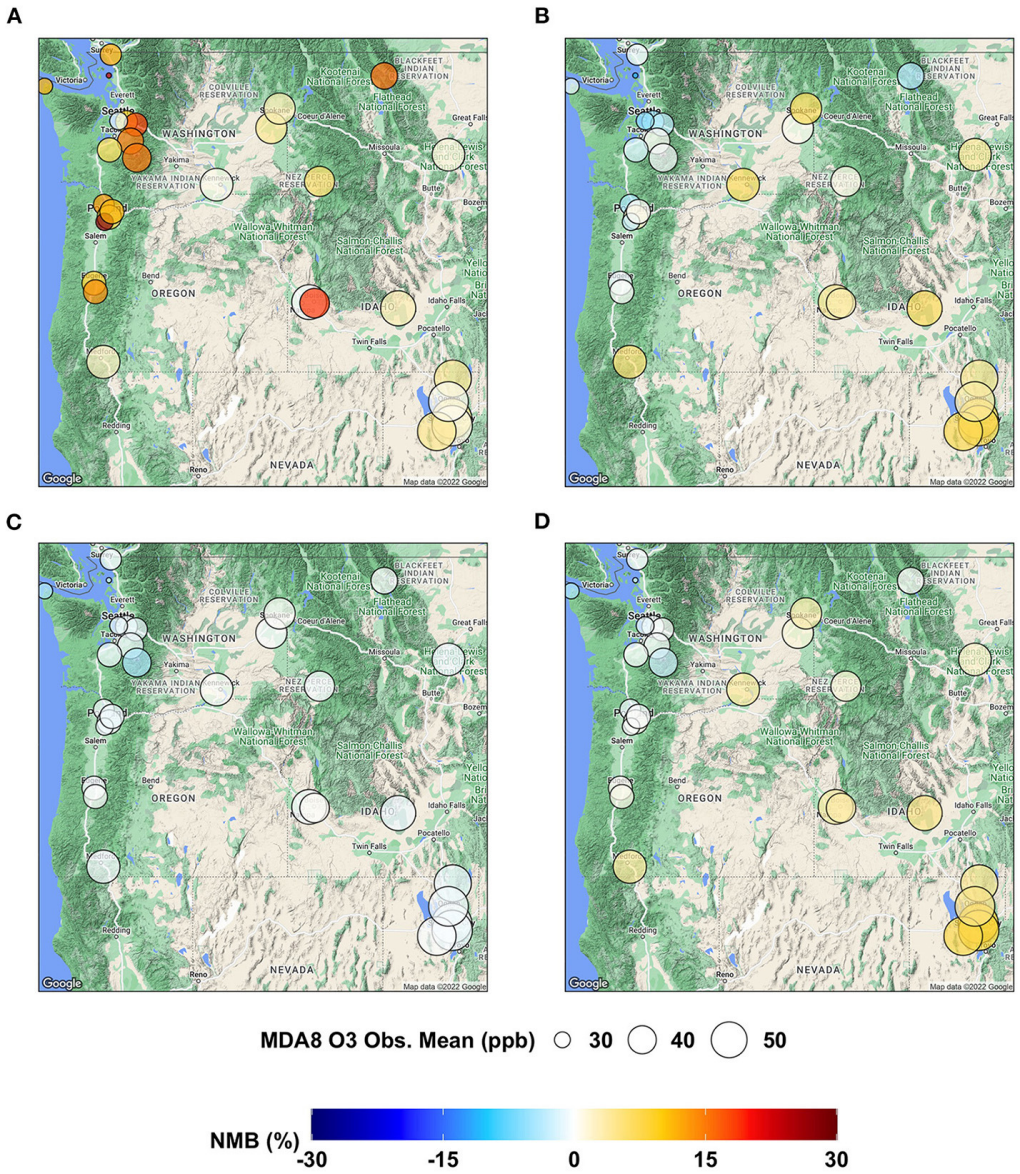
Maps showing NMB of MDA8 O_3_ predictions from **(A)** AIRPACT, **(B)** ML1, **(C)** ML2, and **(D)** ML_opr_O_3_ at the AQS sites throughout the PNW.

**Figure 4:**
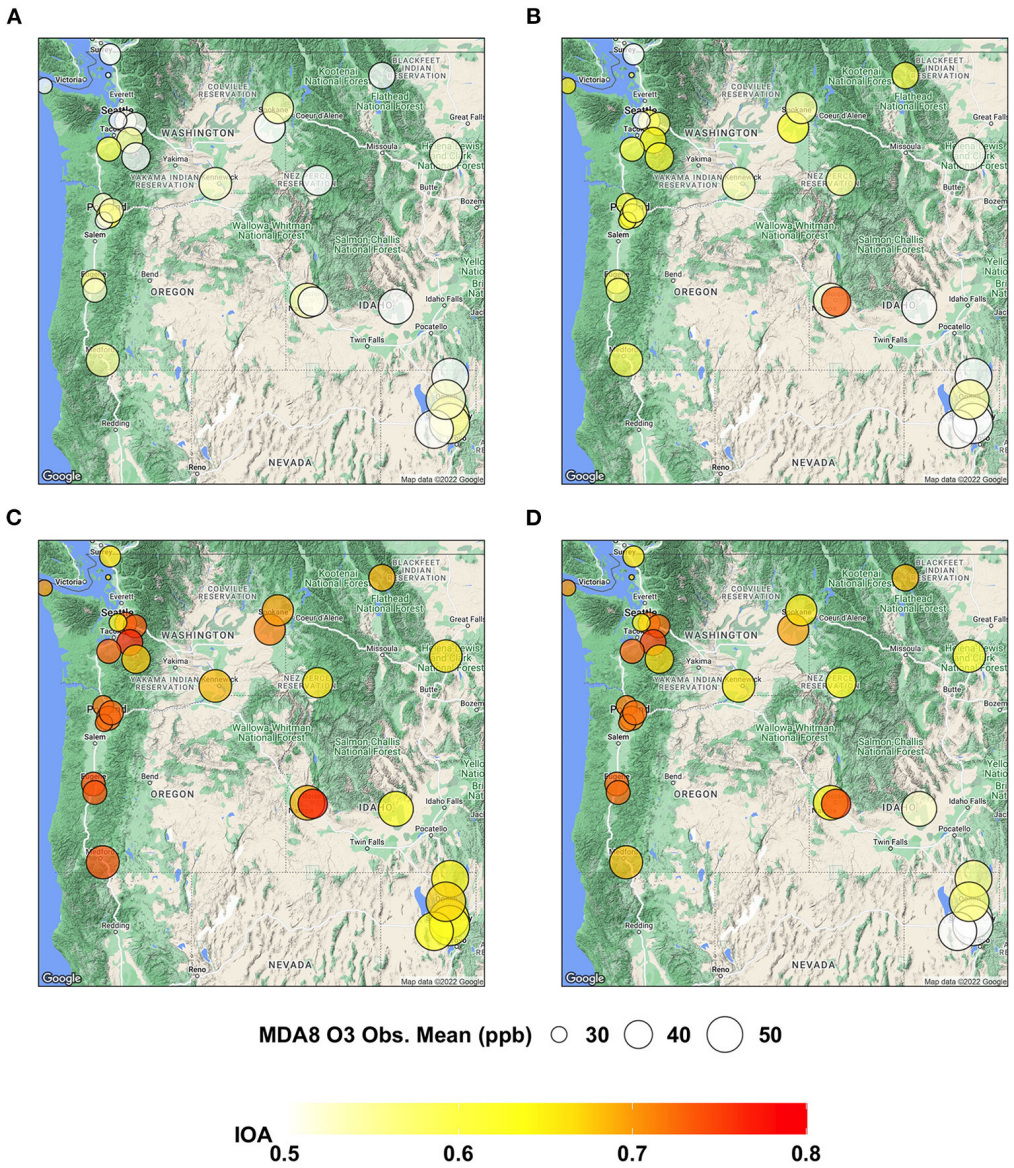
Maps showing IOA of MDA8 O_3_ predictions from **(A)** AIRPACT, **(B)** ML1, **(C)** ML2, and **(D)** ML_opr_O_3_ at the AQS sites throughout the PNW.

To examine the model performance of MDA8 O_3_ at each individual AQS site, we present the spatial distributions of NMB in Figure 3 and those of IOA in Figure 4. AIRPACT tends to over-predict the MDA8 O_3_ during the wildfire seasons, especially along the coast, where the NMB can be up to 28% (see Figure 3A). It is possibly due to the influence of boundary condition and model representation of atmospheric mix layer (Chen et al., 2008). ML1 performs better than AIRPACT and does not over-predict along the coast. The individual AQS site's NMB in ML1 is mostly in the range of −6 to 8%, while that in ML2 is −4% to 0. For ML_opr_O_3_, its NMB is mostly close to the NMB of ML2 except at a few sites (i.e., sites near Salt Lake City, UT) where ML_opr_O_3_ performance is close to ML1. The NME is not shown in the figures, but ML_opr_O_3_ (10 to 14%) and ML2 (8 to 14%) have close performance, and they are better than AIRPACT (12 to 33%) and ML1 (11 to 22%) throughout the AQS sites.

For the site-specific IOA, most ML-based models show higher values than AIRPACT, whose IOA values are mostly below 0.6 (see Figure 4A), because AIRPACT suffers from extremely over-predicted MDA8 O_3_ above 100 ppb and IOA is sensitive to them (Legates and McCabe, 1999). The IOA values of ML_opr_O_3_ are very close to those of ML2, similar to the site-specific NMB.

**Figure 5:**
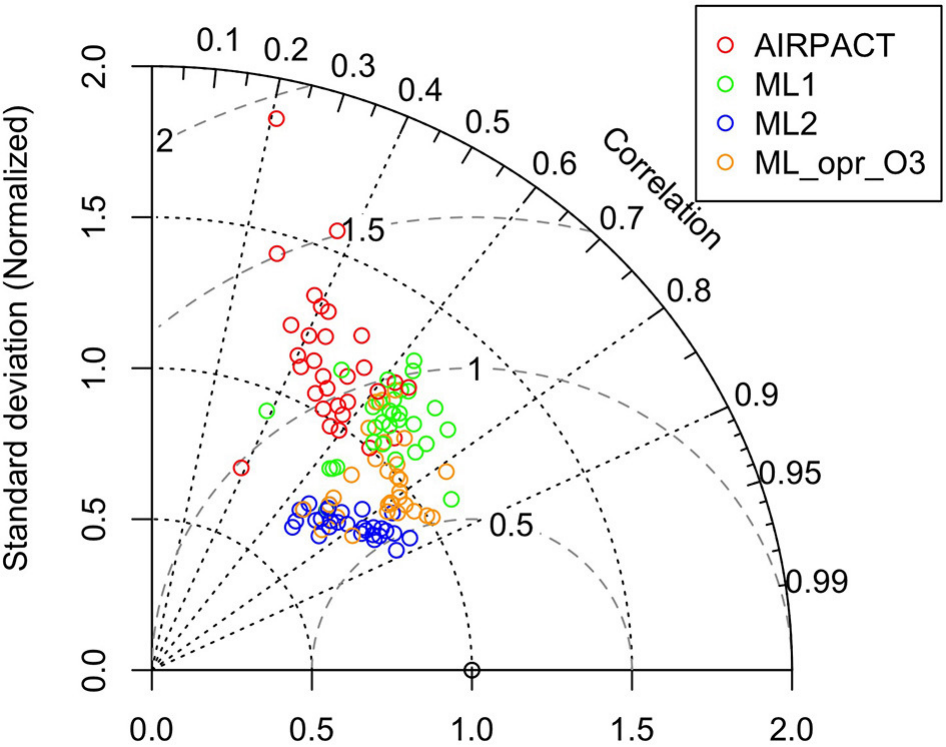
Taylor diagram of MDA8 O_3_ at the AQS sites throughout the PNW. Each circle symbol represents an AQS site, and the red color is for AIRPACT, green for ML1, blue for ML2, and yellow for ML_opr_O_3_. Note that centered RMS difference is proportional to the distance from the point on the x-axis (standard deviation) marked with an open circle.

We also use the Taylor diagram plot in Figure 5 to show the model performance at the individual AQS site. Note that the statistics used in the Taylor diagram are normalized to visualize the difference among models more easily: for example, the SD and centered RMS difference are normalized by dividing them by the observed SD at each AQS site (Taylor, 2001). The Taylor diagram shows that the correlation coefficients of ML2 are within 0.6 and 0.9 and the centered RMS difference values are all within 0.5 and 0.8. While the centered RMS difference of ML1 (0.5 to 1.2) and AIRPACT (0.8 to 2) are worse with a larger site-to-site variation than ML2. However, the normalized SD of ML2 is less than 1, which means the ML2 predictions have less variation than the observations. For ML_opr_O_3_, it is quite like ML2 but the normalized SD is close to 1 for most sites, which means ML_opr_O_3_ is better at capturing the observed variation.

Overall, we find that ML2 predicts the low-MDA8 O_3_ events best, while ML1 predicts the high-MDA8 O_3_ events best. The ML_opr_O_3_ model take an advantage of both ML1 and ML2 by using ML2 model when the ML2 predicted MDA8 O_3_ is lower than 50 ppb and ML1 model for all other cases. The overall ML_opr_O_3_ performance is close to ML2, but it also captures the high-O_3_ events like ML1.

## 4. PM2.5 in 2017 to 2020 wildfire and cold seasons

The PNW region experiences strong seasonal variations of PM2.5 due to distinct sources. For instance, wildfires are the main sources of PM2.5 from May to September, while wood-burning stoves are the main source from November to February. Based on this, our PM2.5 study is separated into the wildfire season (May to September) and cold season (November to February). We use a total of 103 AQS sites for the wildfire season and 104 sites for the cold season, which are available from 2017 to 2020.

A summary of the PM2.5 observations during these seasons is presented in Table 4. The mean PM2.5 concentrations during the wildfire season range from 4.7 to 12 μg m^−3^ while those during the cold season range from 6.9 to 9.2 μg m^−3^. In both seasons, daily PM2.5 concentrations are mostly in the AQI category 1 (AQI_1_; corresponding to Good) and AQI_2_ (Moderate). A large number of wildfires occurred in 2017, 2018 and 2020, leading to 5.0 to 5.9% of monitor-days in the wildfire season experiencing AQI_3_ (unhealthy for sensitive groups) or above. There were few wildfires in 2019, so the mean PM2.5 concentration is particularly low and only 4 AQI_4_ (unhealthy) events occurred during that 2019 wildfire season. The cold season has less variation in PM2.5 concentrations during the 2017 to 2020 period, and experiences significantly fewer unhealthy events (i.e., AQI_3_ and above) than the wildfire season: only 0.1 to 1.1% of monitor-days in the cold season have AQI_3_ or above.

**Table 4:** Summary of the daily PM2.5 observations from two seasons in 2017 to 2020 at AQS sites in the PNW region. Note that daily AQI is computed using PM2.5 only.

**Season**	**Year**	**Mean (μg m^−3^)**	**Percentage and # of monitor-days for each AQI**						
			**1**	**2**	**3**	**4**	**5**	**6**	**Total**
Wildfire season (May to Sep)	2017	11	82.4% (11,442)	11.7% (1,623)	2.9% (409)	2.3% (319)	0.6% (80)	0.1% (16)	13,889
	2018	9.7	83.7% (11,663)	11.2% (1,556)	2.7% (373)	2.3% (321)	0.1% (14)	0 (2)	13,929
	2019	4.7	98.4% (14,144)	1.5% (211)	0.1% (16)	0 (4)	0 (0)	0 (0)	14,375
	2020	12	88.9% (12,556)	6.2% (871)	1.2% (163)	2.1% (296)	1.0% (143)	0.7% (100)	14,129
Cold season (Nov to Feb)	2017	9.1	77.6% (7,997)	21.3% (2,194)	0.9% (97)	0.2% (16)	-	-	10,304
	2018	7.9	82.1%(6,827)	17.7% (1,471)	0.3% (21)	0 (0)	-	-	8,319
	2019	9.2	76.9% (8,843)	22.7% (2,606)	0.4% (51)	0 (3)	-	-	1,1503
	2020	6.9	87.1% (8,647)	12.7% (1,261)	0.1% (14)	0 (0)	-	-	9,922

### 4.1. Evaluating PM2.5 predictions of AIRPACT and ML models in wildfire season

**Figure 6:**
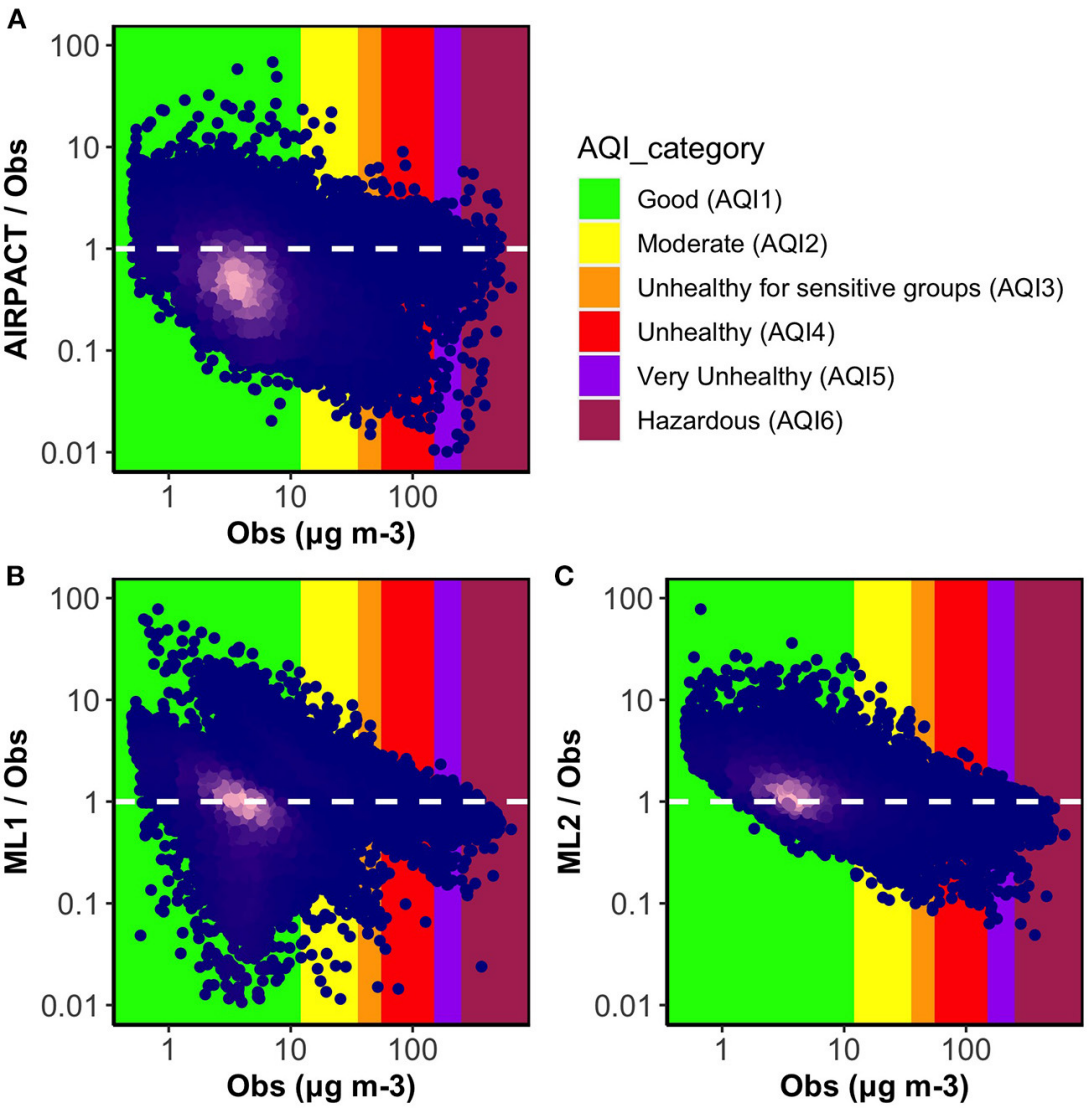
Ratio plots of model predicted daily PM2.5 to observations vs. observations in the wildfire season for three models **(A)** AIRPACT, **(B)** ML1, and **(C)** ML2. The point color of dark blue to bright pink indicates density of the data increasing. The white dashed lines mark the ideal condition (the ratio between model predictions and observations is 1). The ratio below 1 represents the model under-prediction and the ratio above 1 represents the over-prediction.

Similar to the O_3_ evaluation for the ML models, 10-time, 10-fold cross-validation is used to evaluate the ML-based PM2.5 predictions. Because most daily PM2.5 concentrations are below 10 μg m^−3^, the x-axis of ratio plots in Figure 6 uses a log scale. It is clear that PM2.5 predictions are much more scattered, showing severe under-predictions as well as over-predictions than O_3_ predictions shown in Figure 1 for all models. Focusing on the density of data (see the bright pink region in Figure 6A), AIRPACT mostly under-predicts the PM2.5 in the wildfire season: the densest part of the data is below the ratio of 1 in Figure 6A, and Table 5 shows its NMB of −27%. Most of the ML1 and ML2 predictions (bright pink regions in Figures 6B, C) are close to the ratio of 1, and their NMB values (14 and 7.9%) are lower than AIRPACT, although ML1 and ML2 tend to over-predict some low daily PM2.5 concentrations (AQI_1_ and AQI_2_).

**Table 5:** Statistics of the 10-time, 10-fold cross-validations of the daily PM2.5 concentrations during wildfire season from AIRPACT and our ML models.

	**AIRPACT**	**ML1**	**ML2**
R^2^	0.51	0.69	0.72
NMB (%)	−27	14	7.9
NME (%)	59	54	41
IOA	0.67	0.70	0.78

Similar to O_3_, ML2 has a better overall performance than ML1: lower NME (41 vs. 54%) and higher IOA (0.78 vs. 0.70) and higher HSS (0.59 vs. 0.53) are shown in Tables 5, 6. However, unlike O_3_, ML1 does not perform better for high-PM2.5 predictions. The KSS scores from ML1 and ML2 are the same (0.66). The CSI scores for AQI_5_ and AQI_6_ events from ML1 are 0.01 and 0.06 higher than ML2, but the scores for AQI_3_ and AQI_4_ are 0.06 and 0.02 lower than ML2. To reduce the false alarms, we decided to use only ML2 to forecast the daily PM2.5 at the AQS sites in the PNW.

**Table 6:** Forecast verifications of the 10-time, 10-fold cross-validations using AQI computed with only PM2.5 during wildfire season from AIRPACT and our ML models.

		**AIRPACT**	**ML1**	**ML2**
HSS		0.37	0.53	**0.59**
KSS		0.29	**0.66**	**0.66**
CSI	1	0.91	0.91	**0.92**
	2	0.17	0.31	**0.37**
	3	0.08	0.14	**0.20**
	4	0.23	0.36	**0.38**
	5	0.19	**0.24**	0.23
	6	0.30	**0.41**	0.35

The best statistical values are marked with bold fonts.

**Figure 7:**
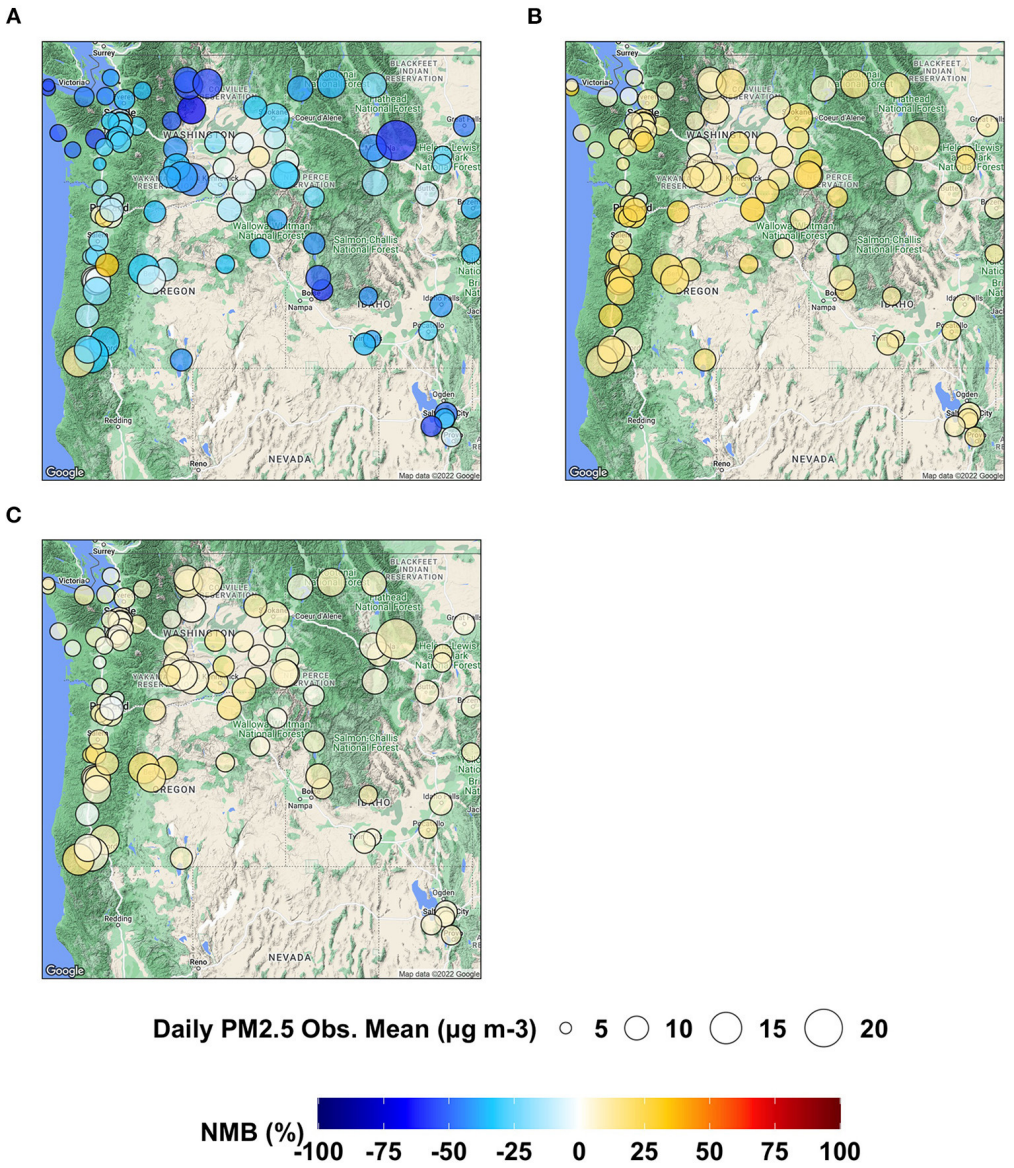
Maps showing NMB of daily PM2.5 predictions from **(A)** AIRPACT, **(B)** ML1, and **(C)** ML2 at the AQS sites throughout the PNW in the wildfire season of 2017 to 2020.

**Figure 8:**
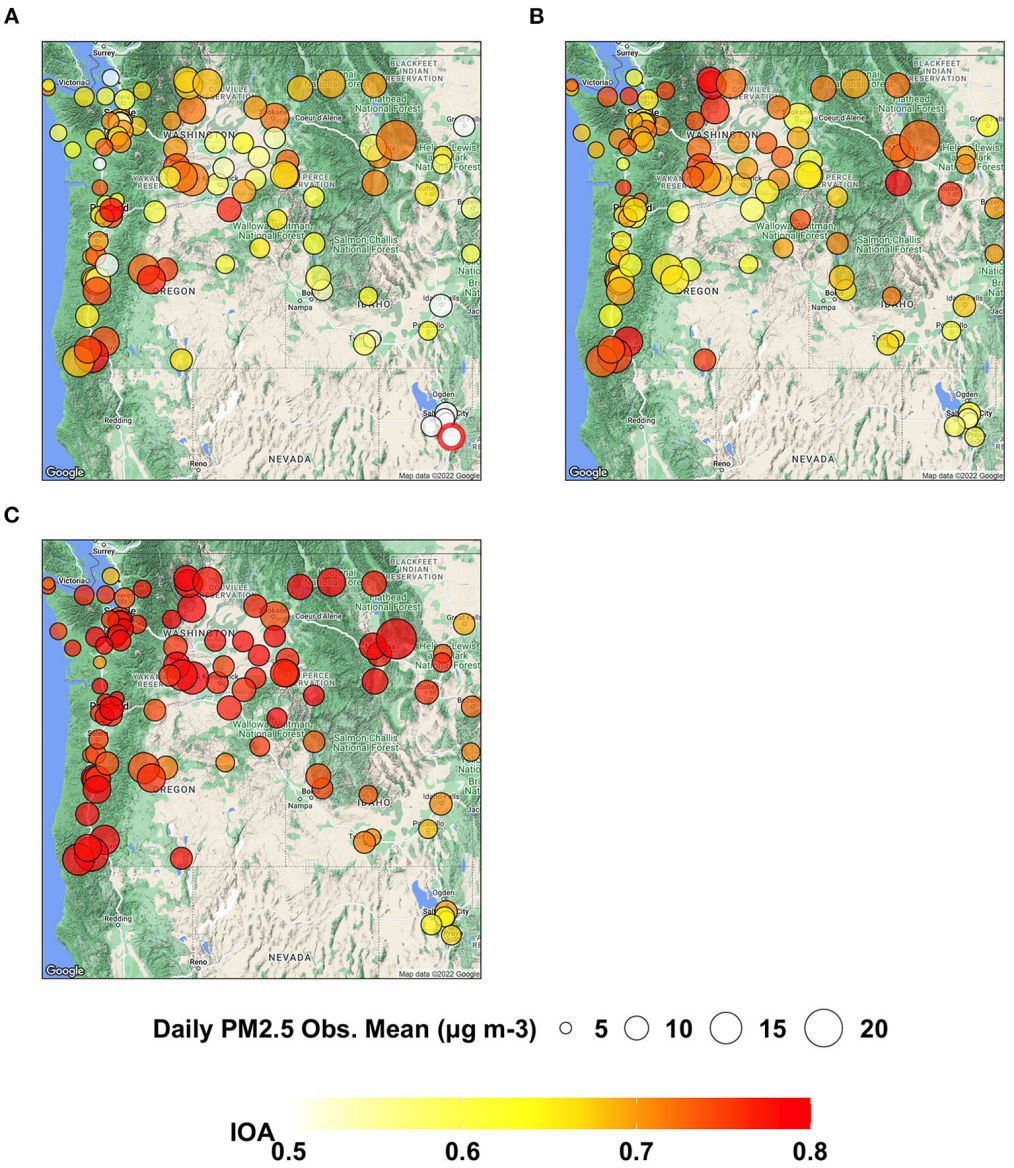
Maps showing IOA of daily PM2.5 predictions from **(A)** AIRPACT, **(B)** ML1, and **(C)** ML2 at the AQS sites throughout the PNW in the wildfire season of 2017 to 2020.

As shown in Figure 7, AIRPACT under-predicts the daily PM2.5 at most AQS sites (94 out of 103 sites) in the PNW, while the ML models tend to over-predict the daily PM2.5. ML2 (−2 to 19%) performs better than ML1 (0 to 32%) because of fewer false alarms. ML2 also has the lowest NME (22 to 60%) than AIRPACT (43 to 103%) and ML1 (33 to 87%) throughout the AQS sites (not shown in the figures). The IOA from AIRPACT in Figure 8A is acceptable except for the AQS sites at the far eastern edge of the model domain. Both ML1 and ML2 show higher IOA than AIRPACT at several sites, but ML2 generally has the highest IOA scores (see Figures 8B, C).

**Figure 9:**
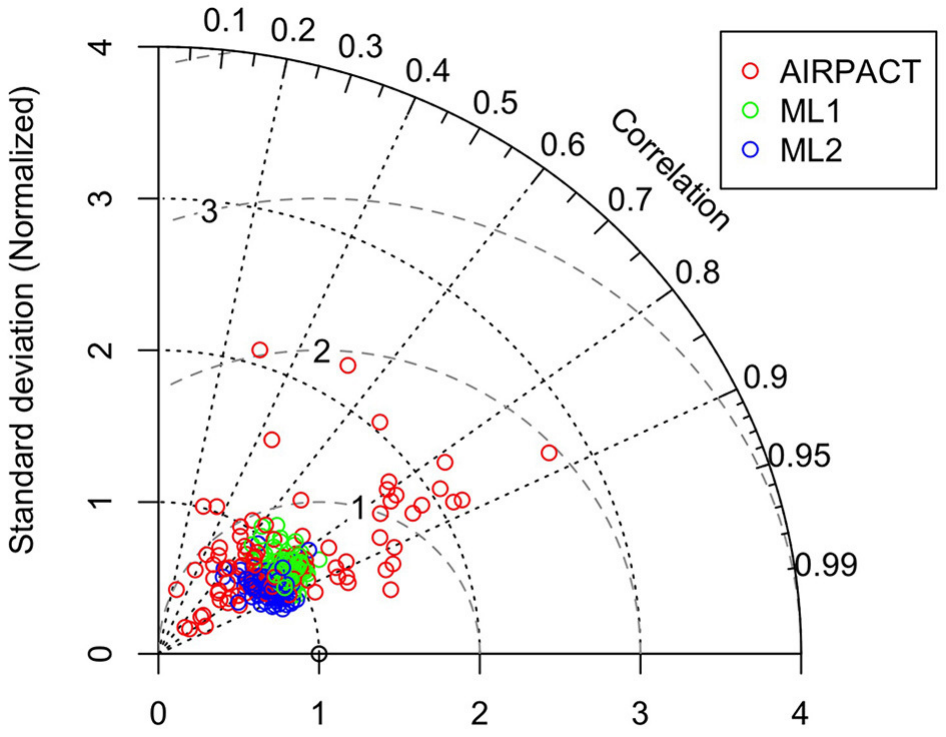
Taylor diagram of wildfire season daily PM2.5. Each circle symbol represents an AQS site, and the red color is for AIRPACT, green for ML1, and blue for ML2. Note that centered RMS difference is proportional to the distance from the point on the x-axis (standard deviation) marked with an open circle.

The Taylor diagram in Figure 9 shows that the AIRPACT performance varies more widely among the 103 AQS sites than ML1 and ML2. The correlation coefficients from AIRPACT range from 0.2 to above 0.9, while both ML predictions are mostly in the range of 0.6 to 0.9. The centered RMS difference values are all within 0.5 to 0.8 for the ML models but AIRPACT has several sites with large centered RMS difference values above 1. Similar to O_3_, the normalized SD of ML1 is close to 1 but that of ML2 is slightly below 1, suggesting the ML2 predictions have less variation than the observations. Figure 9 shows extreme predictions by AIPRACT. For example, the daily PM2.5 concentrations are below 40 μg m^−3^ during wildfire seasons in 2017 to 2020 at Lindon, UT, but AIRPACT predicts several extreme values up to 470 μg m^−3^. The red circle outside the Taylor diagram in Figure 9 is the AQS site represented by the red circle from AIRPACT in Figure 8A, where both ML models perform well.

ML1 and ML2 exhibit better performance for PM2.5 than AIRPACT. However, unlike the case of O_3_ where ML1 shows a significantly better capability to predict high pollution events than ML2, both ML1 and ML2 perform similarly for the high PM2.5 events. Since ML2 preforms noticeably better than ML1 for most PM2.5 levels, we use only ML2 to provide the final PM2.5 forecasts.

### 4.2. Evaluating PM2.5 predictions of AIRPACT and ML models in cold season

There are fewer severe pollution events in the cold season than in wildfire season: only 19 unhealthy events in the cold season of 2017 to 2020; no very unhealthy or hazardous events. AIRPACT during the cold season has the lower NMB (3.4%) and higher NME (67%) than the wildfire season (NMB −27% and NME 59%). Similar to the model performance in the wildfire season, ML1 and ML2 have better statistics than AIRPACT and ML2 shows better performance than ML1 as shown in Supplementary Table 1.

The ratio plot of AIRPACT in Supplementary Figure 3a shows the densest part (bright pink in the figure) is below the 1-to-1 line, which is similar to its predictions during the wildfire season. The low PM2.5 regions show both severe under-prediction and over-prediction but most of the unhealthy events in the red region are mainly under-predicted. Both ML1 and ML2 show better model-to-observation agreements (the scatters in Supplementary Figures 3b, c are closer to the 1-to-1 line), and their NME values (36 and 28%) are much lower than AIRPACT (67%). The IOA, HSS, and KSS scores of ML1 and ML2 are also 0.28 to 0.39 higher than AIRPACT (shown in Supplementary Table 2). In the wildfire season, the CSI_1_ score from AIRPACT (0.91) is comparable to ML models (ML1 0.91, ML2 0.92), but the ML models show significantly better performance at all levels of PM2.5 in the cold season. ML2 has higher CSI_1_ (0.87) and CSI_2_ (0.53) scores than ML1 (0.83 and 0.50), and ML1 has higher CSI_3_ (0.17) and CSI_4_ (0.30) scores than ML2 (0.11 and 0.21).

AIRPACT largely over-predicts the PM2.5 concentrations along the coast in the cold season, where the NMB can be above 100%, while it under-predicts at several inland sites, where the NMB is down to −85% (see Supplementary Figure 4). The NMB values from ML1 and ML2 are mostly in the range of −10 to 20% and −1 to 10%, in respectively, which are better than their performance in the wildfire season. Most of the NME values from two ML models are below 50%, and the AIRPACT can generate extremely high NME, up to 274% (not shown in the figures). Supplementary Figure 5 shows that IOA of AIRPACT is below 0.5 at many AQS sites but both ML models show IOA above 0.5 at most of the AQS sites in the PNW. It clearly shows that our ML models improve forecast performance compared to AIRPACT, and overall ML2 performs better than ML1 at most AQS sites.

Compared to the wildfire season PM2.5 predictions in Figure 9, the centered RMS difference values from AIPRACT are higher than 2 at more sites, and the correlation coefficients decrease from 0.2–0.95 to 0–0.85 in Supplementary Figure 6. The normalized SD values also show a large variation: one value is even above 4 (the red circle outside the Taylor diagram in Supplementary Figure 6), which represents the AQS site in Bellevue, WA. The observed mean PM2.5 at Bellevue is 4.0 μg m^−3^, but the mean prediction from AIRPACT is 14 μg m^−3^, and it predicts several high PM2.5 events up to 67 μg m^−3^. The ML model performance is more stable, and ML2 has more correlation coefficients in the range of 0.8 to 0.9. With the better performance at most sites from ML2 than ML1, the ML2 is also used for the operational PM2.5 forecasts in the cold seasons.

## 5. Summary and conclusions

CTMs are widely used for air quality modeling and forecasting. AIRPACT is a CTM-based operational forecasting system for the PNW, which has been operated for more than a decade. There have been costly efforts to improve AIRPACT forecast capability, but its forecast capability has not been significantly improved, especially for poor air quality events. We developed a ML modeling framework, which we applied successfully to forecast the O_3_ level at Kennewick, WA and used it as a local operational O_3_ forecast. In this study, we expanded the ML modeling framework to predict O_3_ as well as PM2.5 at the AQS sites throughout the PNW. Since April 2020, our ML model has been used for the ensemble-mean 72-h operational air quality forecasts across the AQS sites in the PNW. The Washington State Department of Ecology utilizes our forecasts for their winter forecasts and wildfire smoke forecasts.

There are 30 AQS sites with available O_3_ observations in the wildfire season (May to September) from 2017 to 2020. AIRPACT fails to capture the unhealthy events in the high-O_3_ year, 2017 and 2018, but it performs well in 2019 and 2020. ML1 shows improved predictability for high-O_3_ events, while ML2 shows the best performance for low MDA8 O_3_. The combined approach uses the advantages of the two ML methods and improves the model performance significantly over AIRPACT. The NMB and NME decrease from 7.6 and 18% to 2.6 and 12%, respectively. The statistical parameters, IOA, HSS, KSS, and CSI, are larger than AIRPACT, and the higher CSI_3_ and CSI_4_ scores indicate that the model identifies more high-O_3_ events.

There are 103 AQS sites with available PM2.5 observations during wildfire season and 104 AQS sites during cold season from 2017 to 2020. ML1 and ML2 are trained for two seasons, separately. Both ML models perform much better than AIRPACT. The associated HSS and KSS values for the ML models are 0.22 to 0.39 higher than those for AIRPACT. Compared to AIRPACT and ML1, ML2 has lower NMB and NME and higher IOA in both seasons. The CSI (from CSI_3_ to CSI_6_) values between ML1 and ML2 are quite close, suggesting both models are capable of capturing high-PM2.5 events. Thus, we choose to operate ML2 alone to provide the final PM2.5 predictions.

Our ML modeling framework requires much fewer computing resources than AIRPACT. For example, with the same CPU resources, the ML modeling framework uses one processor to finish training in 40 min with the historical WRF data, and provides up to 30 WRF-member ensemble forecast of O_3_ at 47 AQS sites and PM2.5 at 138 AQS sites throughout the PNW within 1–2 h of CPU time, while AIRPACT needs 120 processors for ~3 h (~360 h of CPU time) to complete the daily operational forecasts.

Overall, the ML modeling framework requires much fewer computational sources and fewer input datasets and provides more reliable air quality forecasts at the selected locations than the CTM-based forecasts, AIRPACT. Our ML models provide more accurate forecasts (most R^2^ >0.7) and captures 70% more high pollution events than AIRPACT. On the basis of the random forest model, we developed this ML modeling framework with preserving the accurate forecasts for the “good” air quality and improving the performance for the “bad” air quality by using the multiple regression model or second-phase random forest.

This study demonstrates the successful application of ML for air quality forecasting and how our ML models can be utilized as a low-cost reliable air quality forecasting system to support regional/local air quality management. Since our ML forecasting system requires previous day's air quality measurement as input, its application is limited to the sites with observations, such as the EPA AQS sites in this study. To expand the utilization in the future, the low-cost sensor measurements (e.g., Purple Air networks) could be employed as the model input to our ML models to provide the air quality forecasts at locations without AQS measurement stations, which would effectively support local air quality management and public awareness.

## Data availability statement

The training datasets for this study can be found in the ROSSENDORF DATA REPOSITORY (RODARE) (https://rodare.hzdr.de/record/2029) (https://doi.org/10.14278/rodare.2029).

## Author contributions

YL and KH conceptualized the overall study. KF implemented the machine learning models, performed the experiments, and validations and analysis with the support of YL, KH, and BL. Datasets used in this study were curated by KF and RD. KF had the lead in writing the manuscript with contributions from YL. All authors revised the final manuscript. All authors contributed to the article and approved the submitted version.
