# Supplementary material for: Machine learning-based ozone and PM2.5 forecasting: Application to multiple AQS sites in the Pacific Northwest
Source: Front Big Data. 2023 Feb 24;6:1124148. doi: 10.3389/fdata.2023.1124148 (PMC9999009; doi:10.3389/fdata.2023.1124148)
Supplement: Supplementary file 1 [file Data_Sheet_1.pdf]

Supplementary Material

1 CODE AVAILABILITY

The GitLab repository can be found at https://gitlab.com/casus_atm_modeling/ml_multi_site/-/tree/kai.

2 SUPPLEMENTARY TABLES AND FIGURES

2.1 Figures

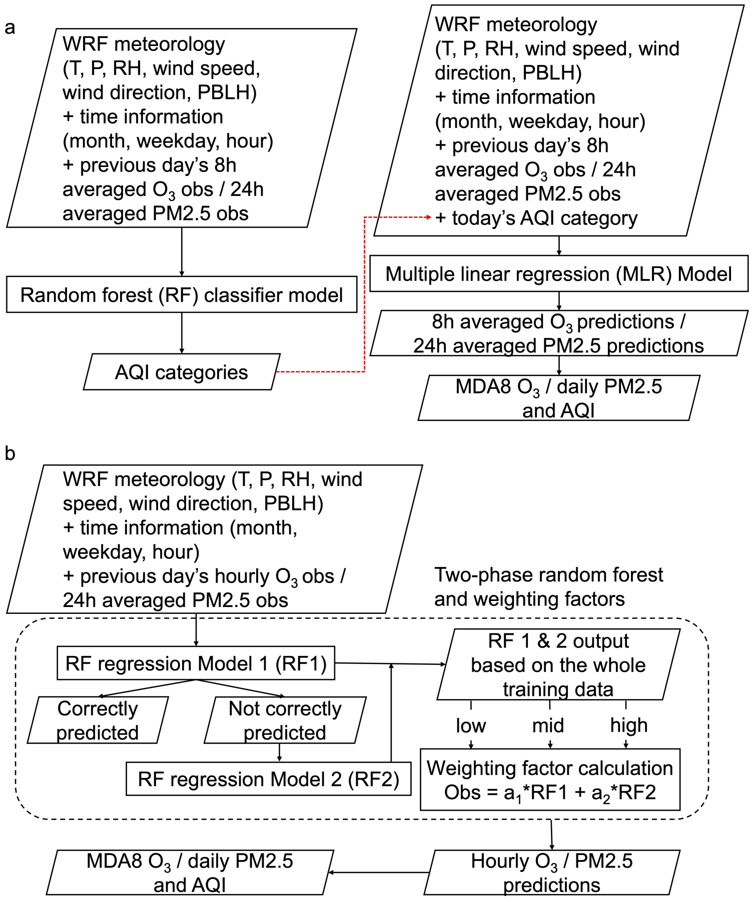

Figure 1. The details of our ML forecast system that consists of two components, ML1 and ML2: (a) ML1 model based on random forest (RF) classifier and multiple linear regression (MLR) models (b) ML2 model based on a two-phase RF regression and weighting factors. (MDA8 O_3: the maximum daily 8-hour moving average O_3).

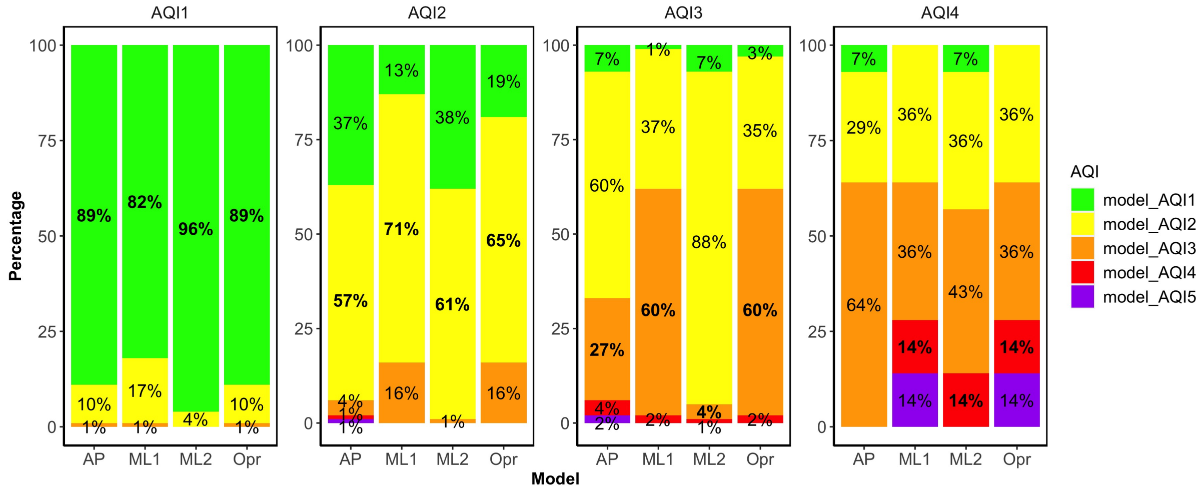

Figure 2. Percentage of model predicted AQI at each observed AQI group. Note that daily AQI is computed using O_3 only.

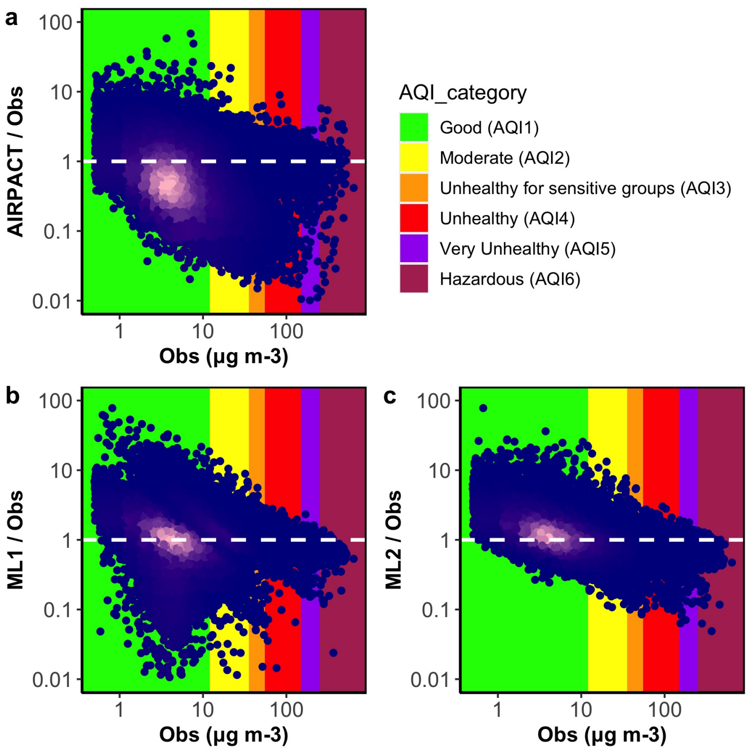

Figure 3. Ratio plots of model predicted daily PM2.5 to observations vs. observations in the cold season for three models (a) AIRPACT, (b) ML1 and (c) ML2. The point color of dark blue to bright pink indicates density of the data increasing. The white dashed lines mark the ideal condition (the ratio between model predictions and observations is 1). The ratio below 1 represents the model under-prediction and the ratio above 1 represents the over-prediction.

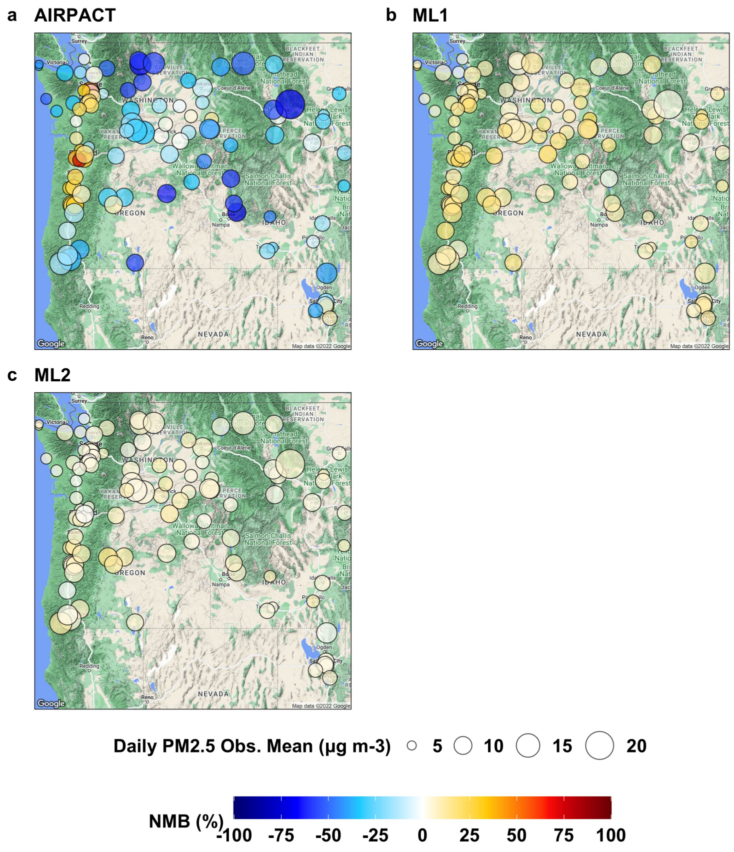

Figure 4. Maps showing NMB of daily PM2.5 predictions from (a) AIRPACT, (b) ML1 and (c) ML2 at the AQS sites throughout the PNW in the cold season of 2017 to 2020.

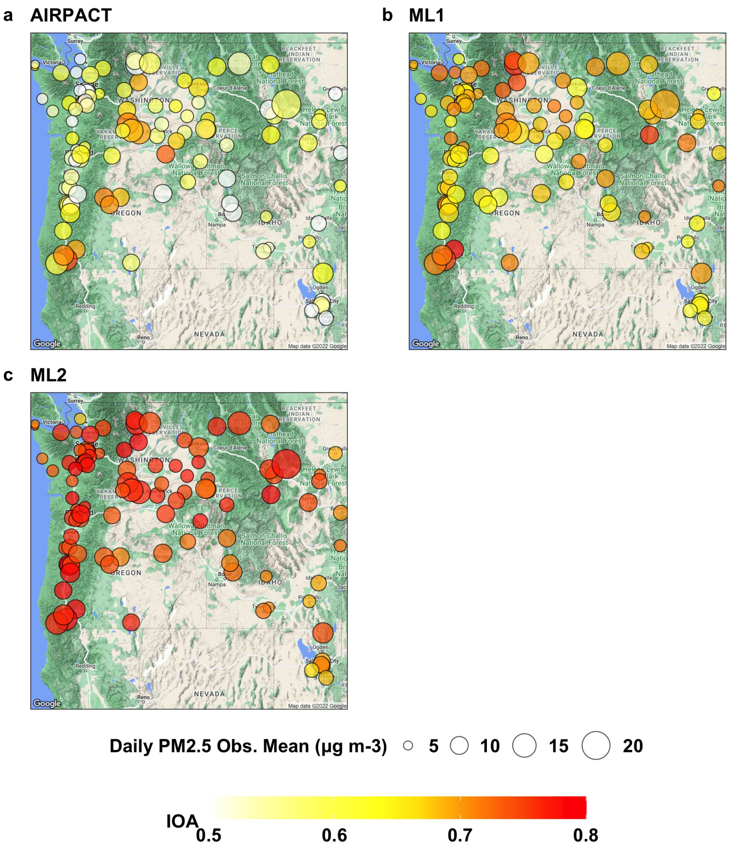

Figure 5. Maps showing IOA of daily PM2.5 predictions from (a) AIRPACT, (b) ML1 and (c) ML2 at the AQS sites throughout the PNW in the cold season of 2017 to 2020.

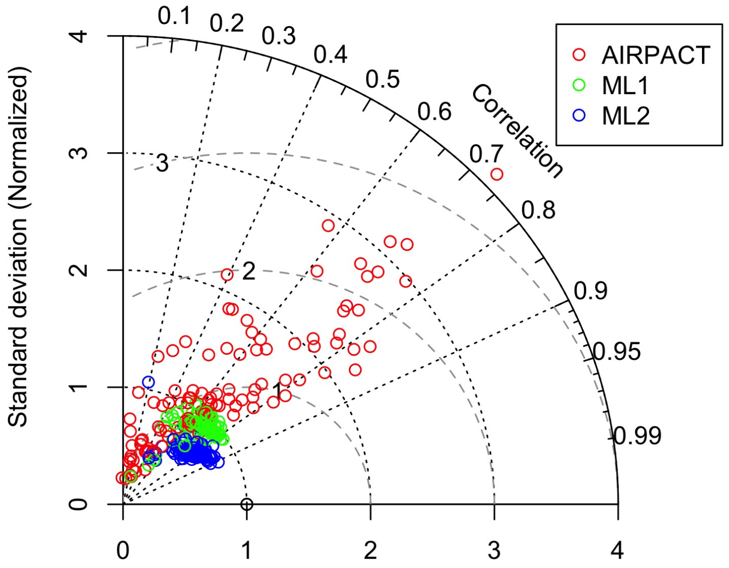

Figure 6. Taylor diagram of cold season daily PM2.5. Each circle symbol represents an AQS site, and the red color is for AIRPACT, green for ML1, and blue for ML2. Note that centered RMS difference is proportional to the distance from the point on the x-axis (standard deviation) marked with an open circle.

2.2 Tables

Table 1. Statistics of the 10-time, 10-fold cross-validations of the daily PM2.5 concentrations during cold season from AIRPACT and our ML models. The best statistical values are marked with bold fonts.

	AIRPACT	ML1	ML2
R^2	0.16	0.58	**0.70**
NMB (%)	3.4	6.7	**2.2**
NME (%)	67	36	**28**
IOA	0.40	0.68	**0.75**

Table 2. Forecast verifications of the 10-time, 10-fold cross-validations using AQI computed with only PM2.5 during cold season from AIRPACT and our ML models. The best statistical values are marked with bold fonts.

		AIRPACT	ML1	ML2
HSS		0.23	0.58	**0.62**
KSS		0.25	**0.64**	0.61
CSI	1	0.74	0.83	**0.87**
	2	0.22	0.50	**0.53**
	3	0.018	**0.17**	0.11
	4	0	**0.30**	0.21
